# Temporal trends and disparities in mortality involving chronic kidney disease and malnutrition among U.S. adults, 1999–2024: a nationwide population-based study

**DOI:** 10.3389/fpubh.2026.1982698

**Published:** 2026-10-12

**Authors:** Yuanhao Chen, Ao Gao, Junman Luo, Yunfeng Hu, Shizhen Liu

**Affiliations:** 1 Department of Nephrology, Jiangmen Central Hospital, Jiangmen, Guangdong, China; 2 Department of Dermatology, The First Affiliated Hospital of Jinan University, Guangzhou, Guangdong, China

**Keywords:** CDC WONDER, chronic kidney disease, malnutrition, mortality and cost effectiveness, public health

## Abstract

**Background:**

Chronic kidney disease (CKD) and malnutrition frequently coexist and are associated with adverse outcomes, yet long-term population-level mortality trends involving both conditions remain poorly characterized. This study examined temporal trends, demographic disparities, and future mortality patterns among U.S. adults with CKD and malnutrition.

**Methods:**

We conducted a nationwide population-based analysis using the Centers for Disease Control and Prevention Wide-ranging Online Data for Epidemiologic Research (CDC WONDER) Multiple Cause of Death database from 1999 to 2024. Adults aged ≥45 years were included when both CKD and malnutrition were documented on the same death certificate, with each condition recorded as either the underlying or a contributing cause of death. Joinpoint regression was used to estimate annual percent changes (APCs) and average annual percent changes (AAPCs). Autoregressive integrated moving average (ARIMA) models were used to project mortality through 2048.

**Results:**

A total of 46,343 deaths involving both CKD and malnutrition were identified. Annual deaths increased from 847 in 1999 to 4,887 in 2024, while the overall AAMR increased from 0.89 to 3.22 per 100,000 population (AAPC, 5.01%; 95% CI, 2.85–7.20). Joinpoint analysis showed an initial decline followed by a marked increase during 2008–2011 and sustained acceleration after 2014. Males had higher AAMRs than females in 2024 (3.57 vs. 2.96 per 100,000). NH Black adults had the highest absolute AAMR (4.75 per 100,000), whereas NH White adults experienced the fastest long-term increase (AAPC, 6.55%; 95% CI, 4.06–9.09). Mortality rose sharply with advancing age, reaching 34.30 per 100,000 among adults aged ≥85 years in 2024. Nonmetropolitan populations had persistently higher mortality than metropolitan populations through 2020. ARIMA projections suggested continued increases in both AAMRs and annual deaths through 2048.

**Conclusion:**

Mortality involving both CKD and malnutrition increased substantially among U.S. adults aged ≥45 years over the past 26 years, with marked disparities by sex, race and ethnicity, age, and urbanization. Earlier nutritional risk identification, strengthened multidisciplinary CKD care, and targeted strategies for high-risk and underserved populations may help mitigate the future mortality burden.

## 1 Introduction

Chronic kidney disease (CKD) is a major global public health concern characterized by progressive loss of kidney function. Its prevalence continues to rise worldwide, affecting approximately 700 million individuals and imposing a substantial burden on morbidity, mortality, and healthcare systems (1, 2). In the United States, CKD affects a considerable proportion of the adult population, with its prevalence increasing markedly with advancing age and among individuals with diabetes, hypertension, and cardiovascular disease (2, 3). As CKD progresses, patients frequently develop a broad spectrum of systemic complications, including cardiovascular disease, anemia, metabolic and mineral disorders, and nutritional abnormalities. Among these complications, malnutrition is particularly important because of its close association with impaired physical function, hospitalization, reduced quality of life, and poor survival (4, 5).

The relationship between CKD and malnutrition is complex and multifactorial. Progressive kidney dysfunction can contribute to reduced dietary intake, anorexia, chronic inflammation, metabolic acidosis, endocrine abnormalities, and enhanced protein catabolism, all of which may promote deterioration of nutritional status (4, 6, 7). In advanced CKD, these disturbances may result in protein-energy wasting (PEW), characterized by depletion of body protein mass and energy reserves (4). PEW is common across the CKD spectrum, with reported prevalence ranging from 11 to 54% among patients with non-dialysis CKD stages 3–5 and an even greater burden among patients receiving maintenance dialysis (5). Conversely, worsening nutritional status may further contribute to muscle wasting, frailty, impaired immune function, and reduced physiological reserve, thereby increasing susceptibility to infection, cardiovascular events, hospitalization, and other adverse outcomes (6–9). These interacting processes may be particularly relevant in older adults, who are more susceptible to both CKD progression and age-related loss of muscle mass and physiological reserve.

Previous studies have consistently demonstrated a strong association between poor nutritional status and adverse outcomes in patients with CKD. Nutritional abnormalities, including hypoalbuminemia, loss of muscle mass, reduced dietary intake, and unfavorable nutritional assessment scores, have been associated with impaired physical function, greater hospitalization burden, and increased mortality, particularly among patients with advanced CKD and those receiving maintenance dialysis (9–11). A recent systematic review and meta-analysis including 29 studies and 11,063 dialysis patients further showed that malnutrition was associated with a 49% higher risk of mortality compared with normal nutritional status (12). These findings have led contemporary clinical guidelines to recommend routine nutritional screening, comprehensive nutritional assessment, and individualized nutrition therapy as integral components of CKD management (13). However, most previous studies have focused on the prevalence and risk factors of malnutrition or PEW, evaluated individual nutritional indicators as predictors of prognosis, or examined the association between nutritional status and mortality within selected clinical or dialysis cohorts (5, 9–12). Although these studies provide important patient-level evidence, their restricted study populations and follow-up periods limit their ability to characterize long-term changes in mortality burden at the population level. Moreover, dialysis populations represent only a subset of individuals with CKD, and evidence derived primarily from these cohorts may not fully reflect mortality patterns across the broader CKD population.

More importantly, national-level temporal trends in mortality involving both CKD and malnutrition remain poorly characterized, particularly across demographic and geographic subgroups. To address this gap, we used the CDC WONDER Multiple Cause of Death database to examine mortality trends and disparities among U.S. adults aged ≥45 years from 1999 to 2024 and to project future mortality patterns. By identifying populations with persistently high or rapidly increasing mortality, this study may provide population-level evidence to inform targeted nutritional surveillance and preventive strategies in CKD care.

## 2 Methods

### 2.1 Data source and study population

This nationwide, population-based study used publicly available mortality data from the Centers for Disease Control and Prevention (CDC) Wide-ranging Online Data for Epidemiologic Research (WONDER) database. Mortality records were obtained from the Multiple Cause of Death Public Use files, which include death certificate data from all 50 U.S. states and the District of Columbia.

The study population included U.S. decedents aged ≥45 years between 1999 and 2024. CKD was identified using the International Classification of Diseases, 10th Revision (ICD-10) code N18 as well as N19, and malnutrition was identified using ICD-10 codes E40–E46. Deaths were included when both CKD and malnutrition were recorded on the same death certificate as either the underlying or a contributing cause of death.

### 2.2 Data extraction

The following variables were extracted from CDC WONDER: year of death, number of deaths, population size, age, sex, race and ethnicity, census region, state of residence, urban–rural classification, crude mortality rate, and age-adjusted mortality rate (AAMR).

Age was categorized as 45–54, 55–64, 65–74, 75–84, and ≥85 years. Race and ethnicity were harmonized into four broad analytical categories: Hispanic or Latino, non-Hispanic (NH) White, NH Black or African American, and NH other racial group. For mortality data from 1999 to 2020, CDC WONDER bridged-race categories were used; the NH other racial group included non-Hispanic American Indian or Alaska Native and Asian or Pacific Islander populations. For 2021–2024, expanded single-race categories were regrouped into the same four analytical categories to improve longitudinal comparability; the NH other racial group included non-Hispanic American Indian or Alaska Native, Asian, Native Hawaiian or Other Pacific Islander, and individuals reporting more than one race. Because the bridged-race and single-race classification systems are not completely equivalent, some residual discontinuity in race-specific estimates across the transition period may remain. Census regions were defined according to U.S. Census Bureau classifications as Northeast, Midwest, South, and West.

Urban–rural status was classified according to the 2013 National Center for Health Statistics Urban–Rural Classification Scheme for Counties and dichotomized into metropolitan and nonmetropolitan areas (14). Because urban–rural mortality data were not available beyond 2020, analyses according to urbanization were restricted to 1999–2020.

### 2.3 Ethical considerations

This study involved secondary analysis of publicly available, de-identified mortality data from the CDC WONDER database. Because the database contains no personally identifiable information, the study did not constitute human subjects research and was exempt from institutional review board approval and informed consent.

### 2.4 Statistical analysis

Annual crude mortality rates and AAMRs were expressed as deaths per 100,000 population. AAMRs were standardized to the 2000 U.S. standard population according to established age-adjustment procedures (15). AAMRs were used for the overall population and analyses stratified by sex, race and ethnicity, census region, and urban–rural residence, whereas crude mortality rates were used for age-specific analyses.

Temporal trends were evaluated using Joinpoint regression, which fits segmented log-linear models to identify statistically significant changes in mortality trends over time (16, 17). For each identified segment, annual percent change (APC) and corresponding 95% confidence intervals (CIs) were estimated. Average annual percent change (AAPC) with 95% CI was calculated to summarize the overall trend across the study period. An APC or AAPC was considered statistically significant when its 95% CI excluded zero.

To characterize future mortality patterns, autoregressive integrated moving average (ARIMA) models were fitted to annual mortality data from 1999 to 2024 using the Box–Jenkins time-series framework (18). Separate models were developed for AAMRs and annual death counts for the overall population, males, and females. Candidate ARIMA (p, d, q) models were selected according to time-series characteristics and model fit, with residual diagnostics used to assess model adequacy. The fitted models were subsequently used to forecast mortality trends through 2048.

Statistical analyses and data processing were performed using R software (version 4.4.0; R Foundation for Statistical Computing, Vienna, Austria). Temporal trend analyses were conducted using the Joinpoint Regression Program (version 5.0; National Cancer Institute, United States) with segmented log-linear regression models. ARIMA forecasting was implemented in R using the forecast package within the Box–Jenkins framework.

All statistical tests were two-sided, and a *p*-value <0.05 was considered statistically significant.

## 3 Results

### 3.1 Overall

Between 1999 and 2024, a total of 46,343 deaths involving chronic kidney disease (CKD) and malnutrition were recorded among U.S. adults aged ≥45 years. The annual number of deaths increased from 847 in 1999 to 4,887 in 2024, representing an increase of 476.98%. The AAMR increased from 0.89 (95% CI, 0.83–0.95) to 3.22 (95% CI, 3.13–3.32) per 100,000 population, corresponding to an AAPC of 5.01% (95% CI, 2.85–7.20; *p* < 0.05) (Table 1).

**Table 1:** Trends in deaths and mortality rates involving chronic kidney disease and malnutrition among U.S. adults aged ≥45 years, 1999–2024.

Characteristic	Deaths			AAMR		
	Deaths_1999	Deaths, end year	Percent.change	AAMR_1999	AAMR, end year	AAPC (95% CI)
Sex						
Both	847	4,887	476.98	0.89 (0.83 to 0.95)	3.22 (3.13 to 3.32)	5.01 (2.85 to 7.20*)
Female	448	2,597	479.69	0.79 (0.72 to 0.86)	2.96 (2.85 to 3.08)	5.27 (−0.02 to 10.84)
Male	399	2,290	473.93	1.09 (0.98 to 1.20)	3.57 (3.42 to 3.72)	4.74 (2.60 to 6.93*)
Census region						
Northeast	100	11,593	11493.00	0.52 (0.42 to 0.63)	21.94 (21.54 to 22.35)	16.53 (11.43 to 21.86*)
Midwest	203	15,934	7749.26	0.87 (0.75 to 1.00)	27.03 (26.61 to 27.46)	15.15 (10.74 to 19.73*)
South	395	26,962	6725.82	1.15 (1.04 to 1.27)	25.35 (25.05 to 25.66)	13.54 (8.67 to 18.62*)
West	149	13,011	8632.21	0.77 (0.65 to 0.90)	20.57 (20.21 to 20.93)	15.24 (9.72 to 21.03*)
Race						
Hispanic	65	293	350.77	1.42 (1.08 to 1.82)	1.89 (1.68 to 2.13)	1.70 (−4.66 to 8.48)
NH Black	244	687	181.56	3.06 (2.67 to 3.45)	4.75 (4.40 to 5.13)	1.87 (−1.94 to 5.82)
NH White	511	3,724	628.77	0.64 (0.58 to 0.69)	3.29 (3.18 to 3.40)	6.55 (4.06 to 9.09*)
NH Other	25	173	592.00	0.94 (0.59 to 1.42)	1.59 (1.36 to 1.85)	2.94 (0.23 to 5.71*)
Age group^#^						
45–54 years	59	110	86.44	0.16 (0.12 to 0.21)	0.27 (0.22 to 0.32)	1.90 (0.09 to 3.74*)
55–64 years	128	296	131.25	0.54 (0.45 to 0.63)	0.71 (0.63 to 0.79)	1.47 (−3.20 to 6.37)
65–74 years	197	848	330.46	1.07 (0.92 to 1.22)	2.39 (2.23 to 2.55)	3.42 (−0.67 to 7.69)
75–84 years	275	1,426	418.55	2.25 (1.98 to 2.52)	7.39 (7.01 to 7.77)	5.10 (3.00 to 7.23*)
85+ years	188	2,207	1073.94	4.53 (3.88 to 5.17)	34.30 (32.87 to 35.73)	7.96 (3.80 to 12.29*)
Urbanization^##^						
Metropolitan	648	3,958	310.80	0.86 (0.79 to 0.92)	2.31 (2.22 to 2.40)	4.40 (1.49 to 7.39*)
Nonmetropolitan	199	891	347.74	1.12 (0.96 to 1.27)	2.43 (2.23 to 2.63)	4.51 (1.79 to 7.30*)

*Deaths, percentage changes in death counts, mortality rates, and average annual percent changes (AAPCs) are presented for the overall population and according to sex, race and ethnicity, age group, and urbanization status. Age-adjusted mortality rates (AAMRs) are expressed per 100,000 population and standardized to the 2000 U.S. standard population. ^#^Age-specific estimates are presented as crude mortality rates per 100,000 population. ^##^Urban–rural analyses were restricted to 1999–2020 because urban–rural mortality data were unavailable after 2020. For urban–rural analyses, the end year was 2020. AAPCs and corresponding 95% confidence intervals (CIs) were estimated using Joinpoint regression.

AAMR, age-adjusted mortality rate; AAPC, average annual percent change; CI, confidence interval; CKD, chronic kidney disease; NH, non-Hispanic.

* indicates a statistically significant AAPC (*P* < 0.05).

Joinpoint regression identified five distinct phases in the overall mortality trend (Figure 1C). The AAMR initially decreased from 1999 to 2008 (APC, −4.58%; 95% CI, −5.80 to −3.34), followed by a sharp increase during 2008–2011 (APC, 24.51%; 95% CI, 7.77–43.85). A non-significant decline occurred from 2011 to 2014 (APC, −7.43%; 95% CI, −17.20 to 3.49). Thereafter, mortality increased significantly from 2014 to 2021 (APC, 15.97%; 95% CI, 14.29–17.67) and continued to rise during 2021–2024 (APC, 6.18%; 95% CI, 3.06–9.39).

**Figure 1:**
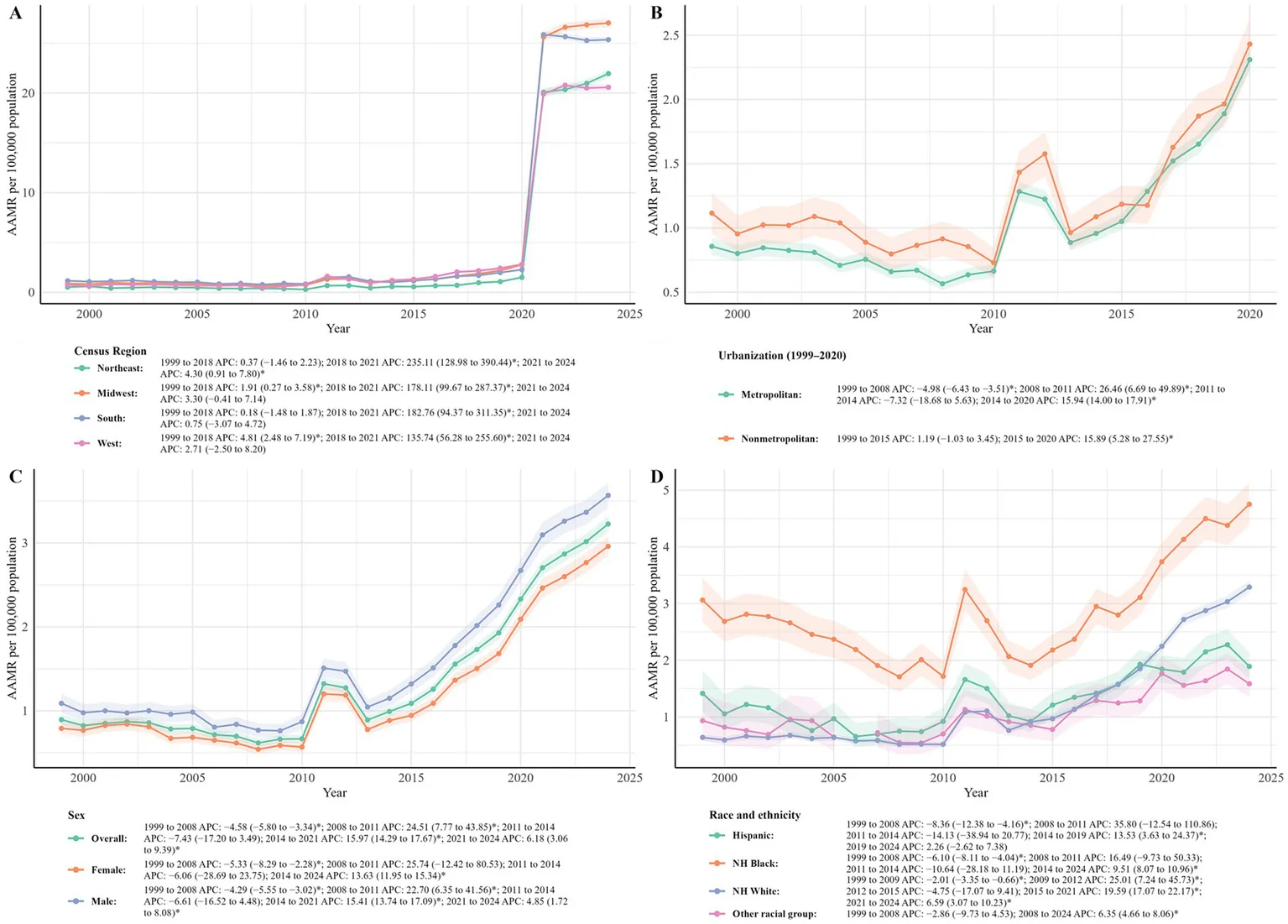
Temporal trends in CKD- and malnutrition-related mortality across demographic and geographic subgroups in the United States, 1999–2024. Annual age-adjusted mortality rates (AAMRs) per 100,000 population are shown according to **(A)** U.S. Census region, **(B)** urbanization status, **(C)** sex, and **(D)** race and ethnicity. Urbanization analyses were restricted to 1999–2020 because more recent urban–rural mortality data were unavailable. Shaded areas represent 95% confidence intervals. Joinpoint regression was used to identify changes in temporal trends, with annual percent changes (APCs) and corresponding 95% confidence intervals reported for each segment. Asterisks indicate statistically significant APCs (*P* < 0.05). The NH other racial group included non-Hispanic American Indian or Alaska Native and Asian or Pacific Islander populations in the 1999–2020 bridged-race data; in the 2021–2024 single-race data, it included non-Hispanic American Indian or Alaska Native, Asian, Native Hawaiian or Other Pacific Islander, and individuals reporting more than one race. CKD, chronic kidney disease; AAMR, age-adjusted mortality rate; APC, annual percent change; NH, non-Hispanic.

### 3.2 Sex-stratified analysis

Sex-specific AAMR trajectories were consistently higher among males than females throughout the study period (Table 1 and Figure 1C). In 1999, the AAMR was 1.09 (95% CI, 0.98–1.20) per 100,000 among males and 0.79 (95% CI, 0.72–0.86) among females. By 2024, the corresponding rates had increased to 3.57 (95% CI, 3.42–3.72) and 2.96 (95% CI, 2.85–3.08) per 100,000, respectively. The AAPC was 4.74% (95% CI, 2.60–6.93) among males and 5.27% (95% CI, −0.02 to 10.84) among females.

Among males, mortality declined from 1999 to 2008 (APC, −4.29%), increased during 2008–2011 (APC, 22.70%), and declined again during 2011–2014 before increasing significantly from 2014 to 2021 (APC, 15.41%) and 2021 to 2024 (APC, 4.85%). Females showed a similar early decline from 1999 to 2008 (APC, −5.33%), followed by fluctuations through 2014 and a sustained increase from 2014 to 2024 (APC, 13.63%).

### 3.3 Age group-stratified analysis

Age-specific trends were assessed using crude mortality rates (Table 1 and Figures 2, 3). Mortality increased progressively with advancing age. In 2024, the crude mortality rates were 0.27, 0.71, 2.39, 7.39, and 34.30 per 100,000 population among adults aged 45–54, 55–64, 65–74, 75–84, and ≥85 years, respectively.

**Figure 2:**
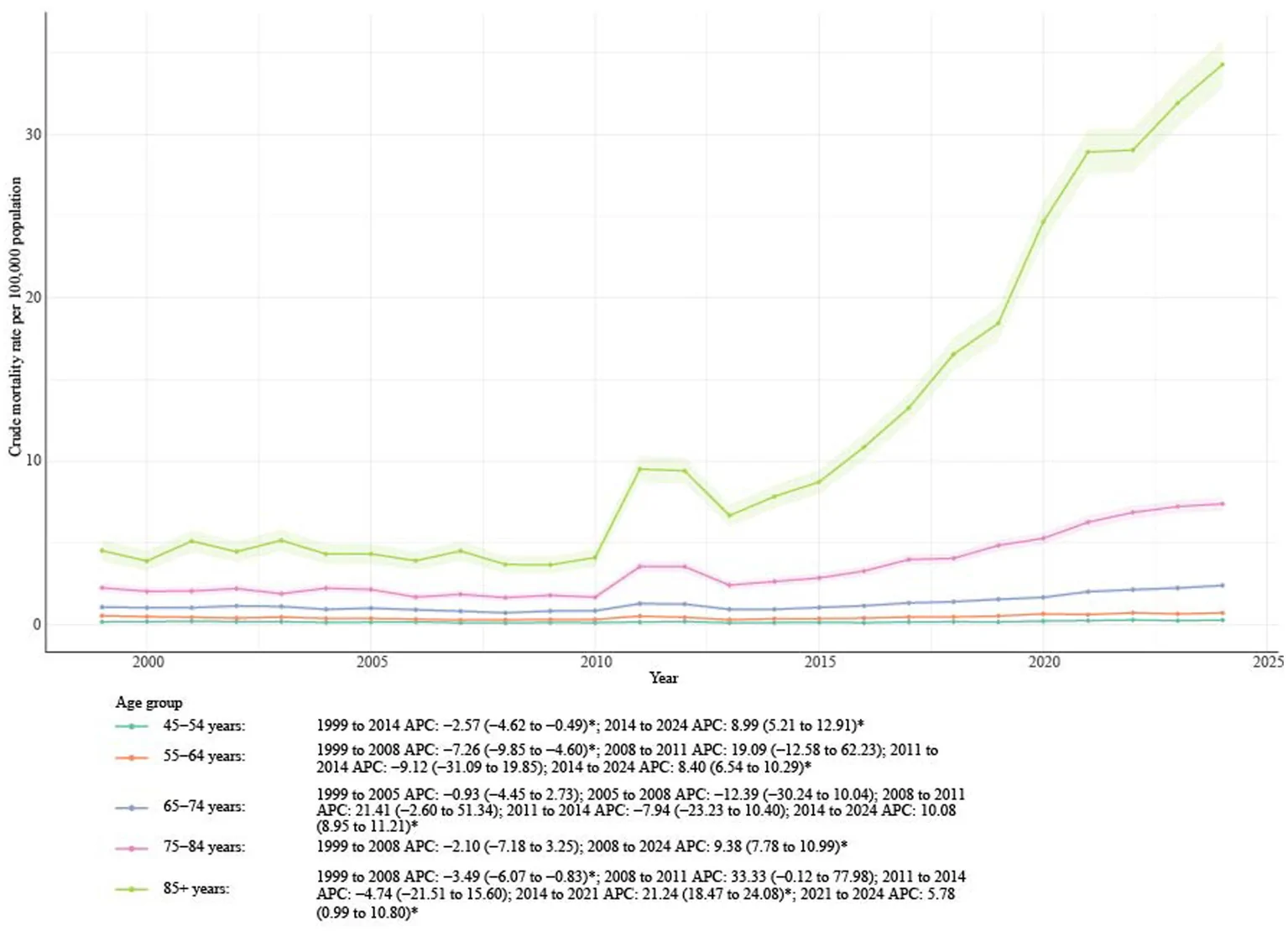
Age-specific trends in CKD- and malnutrition-related mortality in the United States, 1999–2024. Annual age-specific crude mortality rates per 100,000 population are presented for adults aged 45–54, 55–64, 65–74, 75–84, and ≥85 years. Shaded areas represent 95% confidence intervals. Joinpoint regression was used to characterize temporal changes within each age group, with APCs and corresponding 95% confidence intervals reported for each segment. Asterisks indicate statistically significant APCs (*P* < 0.05). CKD, chronic kidney disease; APC, annual percent change.

**Figure 3:**
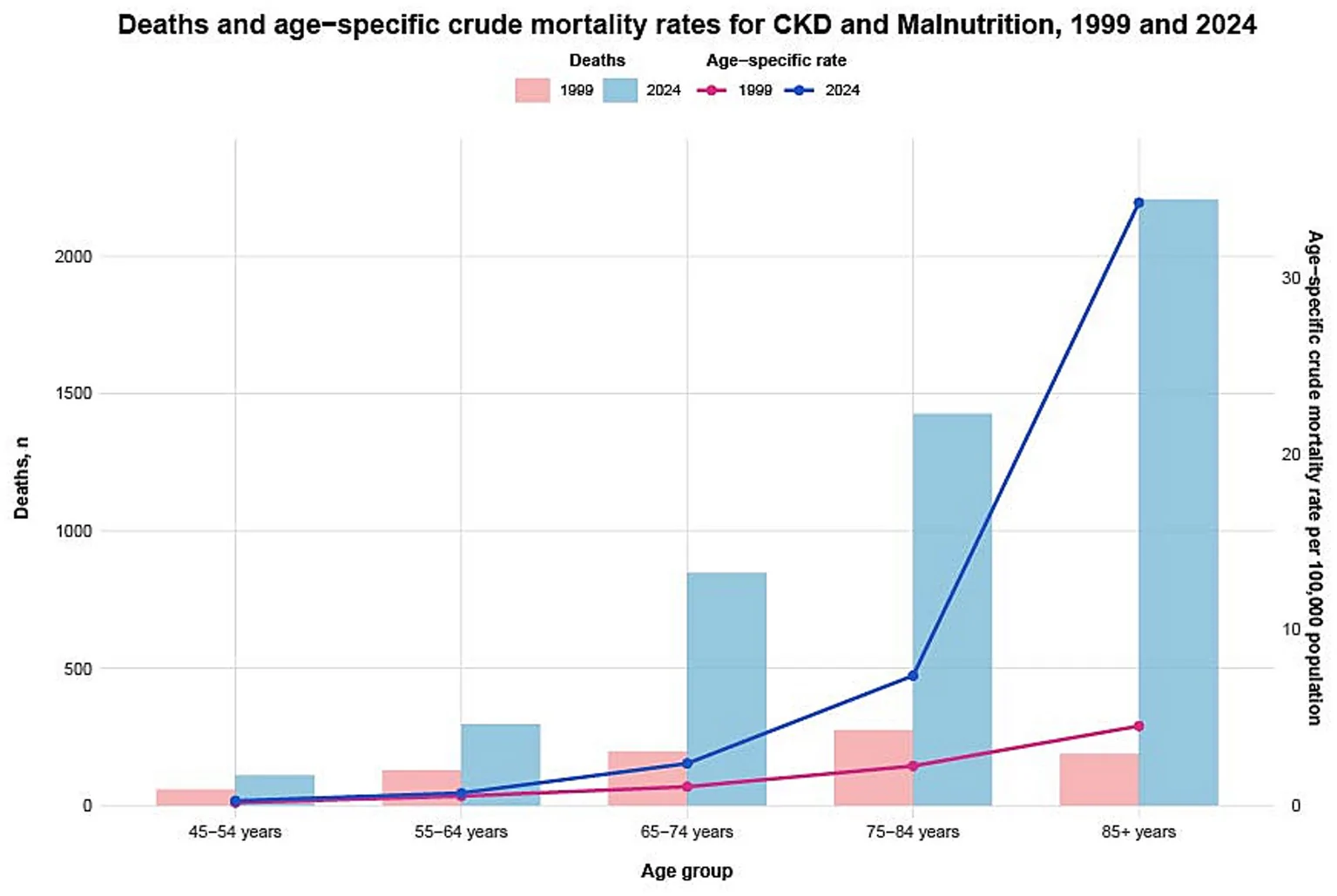
Death counts and age-specific crude mortality rates for CKD- and malnutrition-related mortality by age group in the United States, 1999 and 2024. Bars represent the number of deaths in 1999 and 2024, and lines represent the corresponding age-specific crude mortality rates per 100,000 population. Results are presented for adults aged 45–54, 55–64, 65–74, 75–84, and ≥85 years. The left y-axis indicates the number of deaths, and the right y-axis indicates the age-specific crude mortality rate. CKD, chronic kidney disease.

Among adults aged 45–54 years, the mortality rate increased from 0.16 in 1999 to 0.27 per 100,000 in 2024, with an AAPC of 1.90% (95% CI, 0.09–3.74). The rate declined during 1999–2014 and subsequently increased significantly during 2014–2024 (APC, 8.99%).

Among adults aged 55–64 years, the mortality rate increased from 0.54 to 0.71 per 100,000, with an AAPC of 1.47% (95% CI, −3.20 to 6.37). After an initial decline during 1999–2008, mortality increased significantly from 2014 to 2024 (APC, 8.40%).

For adults aged 65–74 years, the mortality rate increased from 1.07 to 2.39 per 100,000, with an AAPC of 3.42% (95% CI, −0.67 to 7.69). A sustained increase was observed after 2014 (APC, 10.08%).

Adults aged 75–84 years experienced a larger increase, from 2.25 to 7.39 per 100,000, corresponding to an AAPC of 5.10% (95% CI, 3.00–7.23). Mortality increased significantly from 2008 to 2024 (APC, 9.38%).

The highest mortality burden was observed among adults aged ≥85 years. Deaths increased from 188 in 1999 to 2,207 in 2024, while the crude mortality rate increased from 4.53 to 34.30 per 100,000. This group had the highest AAPC (7.96%; 95% CI, 3.80–12.29). Mortality increased particularly rapidly during 2014–2021 (APC, 21.24%) and remained significantly elevated during 2021–2024 (APC, 5.78%).

The comparison between 1999 and 2024 further demonstrated that both death counts and crude mortality rates increased progressively with age, with the largest absolute increases observed among adults aged 75–84 and ≥85 years (Figure 3).

### 3.4 Race-stratified analysis

Substantial racial and ethnic differences in mortality were observed over the study period (Table 1 and Figure 1D). NH Black adults had the highest AAMR, increasing from 3.06 (95% CI, 2.67–3.45) in 1999 to 4.75 (95% CI, 4.40–5.13) per 100,000 in 2024. The corresponding AAPC was 1.87% (95% CI, −1.94 to 5.82).

Among NH White adults, the AAMR increased from 0.64 to 3.29 per 100,000, with an AAPC of 6.55% (95% CI, 4.06–9.09). Joinpoint regression showed an early decline from 1999 to 2009, followed by a rapid increase during 2009–2012, a short decline during 2012–2015, and significant increases during 2015–2021 (APC, 19.59%) and 2021–2024 (APC, 6.59%).

Among NH Black adults, mortality declined during 1999–2008 and subsequently increased significantly from 2014 to 2024 (APC, 9.51%). Hispanic adults showed a decline during 1999–2008, followed by a significant increase during 2014–2019 (APC, 13.53%). Among adults in the NH other racial group, mortality increased significantly from 2008 to 2024 (APC, 6.35%).

### 3.5 Census region-stratified analysis

This subsection will be finalized after correction of the 2021–2024 Census-region data.

Across the four census regions, geographic differences in CKD- and malnutrition-related mortality were observed during the available study period (Figure 1A and Table 1). Region-specific AAMRs and Joinpoint trajectories will be reported after the corrected Multiple Cause of Death data for 2021–2024 are incorporated.

### 3.6 Urbanization-stratified analysis

Urban–rural disparities in AAMR were evident throughout the available observation period (1999–2020), with nonmetropolitan areas maintaining higher mortality rates than metropolitan areas (Figure 1B and Table 1).

In metropolitan areas, deaths increased from 648 in 1999 to 2,822 in 2020, while the AAMR increased from 0.86 to 2.31 per 100,000, corresponding to an AAPC of 4.40% (95% CI, 1.49–7.39). Mortality declined during 1999–2008, increased sharply during 2008–2011, declined during 2011–2014, and subsequently increased significantly from 2014 to 2020 (APC, 15.94%).

In nonmetropolitan areas, deaths increased from 199 to 587, while the AAMR increased from 1.12 to 2.43 per 100,000, with an AAPC of 4.51% (95% CI, 1.79–7.30). Rates remained relatively stable through 2015, followed by a significant increase during 2015–2020 (APC, 15.89%).

### 3.7 Forecasted mortality trends

ARIMA forecasting showed continued increases in both AAMRs and annual deaths involving CKD and malnutrition through 2048 (Figure 4). The projected AAMR increased steadily for the overall population and for both sexes, with males maintaining higher predicted mortality rates than females throughout the forecast period.

**Figure 4:**
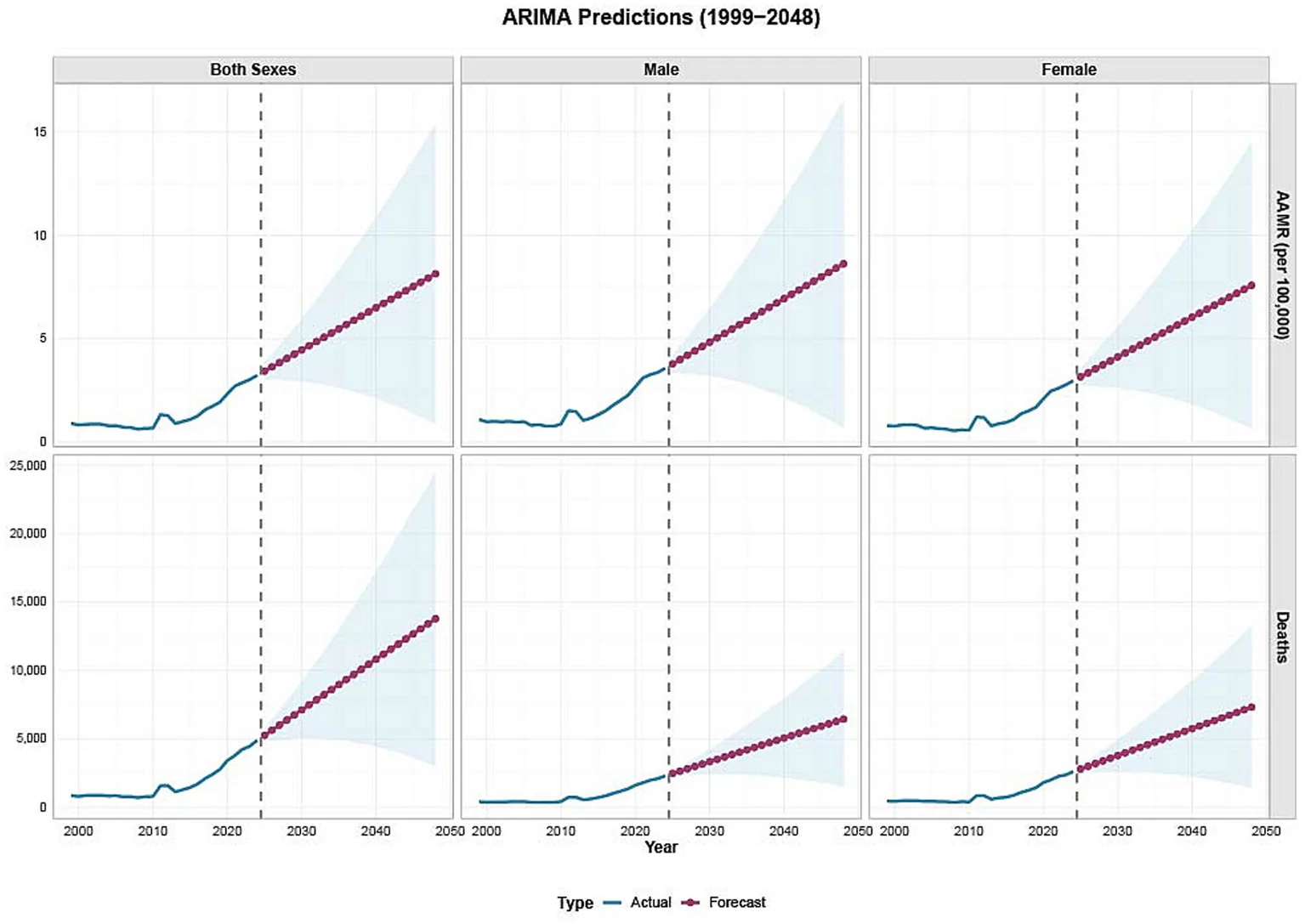
Observed and projected CKD- and malnutrition-related mortality in the United States, 1999–2048. Autoregressive integrated moving average (ARIMA) models were used to project age-adjusted mortality rates (AAMRs) and annual death counts through 2048 for the overall population, males, and females. The upper panels show AAMRs per 100,000 population, and the lower panels show annual numbers of deaths. Solid lines represent observed values through 2024, points represent model-based forecasts for 2025–2048, and shaded areas represent prediction intervals. The vertical dashed line indicates the transition between the observed and forecast periods. CKD, chronic kidney disease; AAMR, age-adjusted mortality rate; ARIMA, autoregressive integrated moving average.

Similarly, the projected number of deaths increased progressively in the overall, male, and female populations. Prediction intervals widened with increasing forecast horizon, indicating greater uncertainty in long-term estimates. Nevertheless, the central forecasts consistently indicated a sustained upward trajectory in CKD- and malnutrition-related mortality through 2048.

## 4 Discussion

Using nationwide mortality data from the CDC WONDER Multiple Cause of Death database, this study identified a sustained increase in mortality involving both chronic kidney disease (CKD) and malnutrition among U.S. adults aged ≥45 years from 1999 to 2024, with a pronounced acceleration after 2014. The burden was unevenly distributed across demographic and geographic subgroups. Males and non-Hispanic (NH) Black adults experienced comparatively high mortality, whereas NH White adults showed the fastest long-term increase. Mortality rose sharply with advancing age and was particularly pronounced among adults aged ≥85 years, while nonmetropolitan populations experienced a persistently higher burden than metropolitan populations. Time-series projections further suggested that both mortality rates and annual deaths may continue to increase if recent patterns persist. Collectively, these findings indicate that mortality involving CKD and malnutrition represents a growing public health challenge characterized by substantial demographic and geographic disparities.

The sustained upward trend is biologically and clinically plausible given the close interaction between kidney dysfunction and nutritional deterioration. Malnutrition in CKD is rarely attributable to inadequate dietary intake alone. Progressive loss of kidney function may be accompanied by anorexia, chronic inflammation, metabolic acidosis, endocrine disturbances, and enhanced protein catabolism, collectively contributing to protein-energy wasting (PEW) (4, 7). Malnutrition and inflammation may further reinforce one another, resulting in progressive depletion of body protein stores, impaired immune function, and diminished physiological reserve; this malnutrition–inflammation complex has long been associated with adverse outcomes in advanced kidney disease (19). These processes suggest that the coexistence of CKD and malnutrition may represent a broader state of metabolic, inflammatory, and functional vulnerability rather than the simple coexistence of two independent conditions.

Our findings should also be interpreted in the context of previously reported national mortality trends for CKD and malnutrition individually. A CDC WONDER-based study of U.S. adults aged ≥65 years reported an overall increase in CKD-related mortality from 1999 to 2020 (20), whereas a separate national analysis of malnutrition-related mortality among adults aged ≥65 years found an early decline followed by a pronounced increase beginning in 2013 (21). The trajectory observed in our study partially overlaps with these broader patterns, particularly the marked increase in mortality involving both CKD and malnutrition after 2014. Thus, the post-2014 acceleration should not be interpreted as uniquely specific to the co-occurrence of CKD and malnutrition. Rather, it occurred within a broader period of increasing CKD-related and, particularly, malnutrition-related mortality. Nevertheless, unlike previous studies examining either condition separately, our analysis specifically quantified deaths in which both CKD and malnutrition were documented on the same death certificate. Direct quantitative comparisons should be interpreted cautiously because the prior studies were restricted to adults aged ≥65 years, whereas the present study included adults aged ≥45 years.

The temporal trajectory also warrants attention. Mortality declined during the early study period, increased sharply during 2008–2011, temporarily attenuated, and subsequently entered a sustained period of rapid increase after 2014. Because the present analysis is ecological, the factors responsible for these inflection points cannot be determined directly. Population aging may have contributed to the increase in the absolute number of deaths, although it cannot directly explain the rise in age-adjusted mortality rates. The increasing burden of multimorbidity, frailty, sarcopenia, and nutritional vulnerability among individuals living with CKD may have contributed to the observed mortality patterns (22, 23). Changes in the recognition and documentation of malnutrition should also be considered. In 2012, the Academy of Nutrition and Dietetics and the American Society for Parenteral and Enteral Nutrition introduced consensus recommendations to standardize the identification and documentation of adult malnutrition (24), which may have contributed to greater clinical recognition in subsequent years. However, U.S. cause-of-death data have used ICD-10 throughout the present study period, beginning in 1999 (25), and the available data do not establish that a discrete mortality-coding change caused the post-2014 inflection. Therefore, the observed acceleration may reflect a combination of demographic, clinical, and documentation-related factors, but their individual contributions cannot be quantified using CDC WONDER data.

The increase observed around 2020 may also have been influenced by the COVID-19 pandemic. Patients with advanced kidney disease, particularly those receiving dialysis, experienced substantial excess mortality during the early pandemic (26), and CKD was associated with increased mortality among patients with COVID-19 in a systematic review and meta-analysis (27). In addition to the direct effects of SARS-CoV-2 infection, pandemic-related healthcare disruption and health-system strain may have affected access to routine CKD management, nutritional assessment, and multidisciplinary care. However, the present analysis did not separately determine whether COVID-19 was documented on the death certificates included in this study, and CDC WONDER does not provide individual-level information regarding infection timing, healthcare disruptions, or treatment changes. Therefore, although the pandemic may have contributed to the increase observed around 2020, its specific contribution cannot be determined from these data.

Sex disparities remained evident throughout the study period, with males consistently exhibiting higher AAMRs than females. Sex differences in CKD epidemiology and outcomes have been increasingly recognized. Although women may have a higher prevalence of some stages of CKD, men generally demonstrate greater progression toward kidney failure and higher risks of several CKD-related complications (28). Recent evidence has further shown sex-related differences in cardiovascular complications and mortality across levels of kidney function (29), while a large meta-analysis demonstrated that associations between kidney dysfunction and mortality may differ between men and women (30). These differences are likely multifactorial and may involve hormonal influences, cardiovascular risk profiles, body composition, health behaviors, and patterns of healthcare use. In the context of malnutrition, differences in skeletal muscle mass and responses to catabolic stress may further modify mortality risk. Importantly, mortality increased substantially in both sexes, indicating that nutritional vulnerability should be considered across the entire CKD population rather than being regarded as a sex-specific problem.

Racial and ethnic disparities were another prominent finding. NH Black adults experienced the highest absolute mortality burden, whereas NH White adults showed the most rapid long-term increase. The disproportionately high mortality among NH Black adults is consistent with longstanding racial inequities in the incidence, progression, treatment, and outcomes of kidney disease in the United States. Previous studies have documented higher mortality among Black adults with CKD and emphasized that such disparities arise from complex interactions among socioeconomic conditions, healthcare access, structural disadvantage, and differences in comorbidity burden (31–33). City-level analyses have similarly demonstrated persistent Black–White inequities in kidney disease mortality across the United States (34). Hispanic adults also experienced a significant increase in mortality during 2014–2019, while adults in the NH other racial group showed a significant increasing trend from 2008 to 2024. These findings indicate that the increasing burden of mortality involving CKD and malnutrition was not confined to NH Black or NH White populations. However, interpretation of the NH other racial group requires caution because it combines multiple populations, including American Indian or Alaska Native, Asian, Native Hawaiian or Other Pacific Islander, and individuals reporting more than one race. This aggregation may obscure important differences among smaller racial subgroups. Future studies incorporating more detailed racial and ethnic classifications and individual-level clinical and socioeconomic information are needed to better understand the mechanisms contributing to these disparities.

Nutritional vulnerability may further amplify these inequalities. Effective nutritional management in CKD requires patients to maintain sufficient energy and nutrient intake while navigating disease-specific dietary restrictions, which may be particularly difficult in the presence of food insecurity. Among U.S. adults with CKD, food insecurity has been associated with an increased risk of progression to kidney failure (35). A systematic review further highlighted the relationship between food insecurity, kidney disease, and adverse health outcomes (36). Limited access to affordable nutritious food may therefore interact with CKD-related dietary restrictions, low income, multimorbidity, and fragmented healthcare to accelerate nutritional deterioration. From a public health perspective, this suggests that CKD-associated malnutrition cannot be addressed solely through individual dietary advice; upstream socioeconomic and nutritional determinants also require consideration.

Urban–rural disparities provide further evidence that mortality involving CKD and malnutrition is influenced by healthcare accessibility and place of residence. Nonmetropolitan populations consistently experienced higher mortality than metropolitan populations during the period for which urbanization data were available. Rural communities frequently face longer travel distances, shortages of nephrologists and renal dietitians, delayed specialist referral, and limited availability of multidisciplinary kidney care (37). These barriers may be particularly consequential for patients with nutritional deterioration, which requires repeated screening and longitudinal management rather than a single clinical encounter. More broadly, disparities in the burden and care of CKD have been linked to differences in socioeconomic resources, preventive healthcare access, and continuity of care (38). The urban–rural gap observed in this study should therefore be considered not merely a geographic variation but an important manifestation of health inequity.

Age was one of the strongest determinants of mortality burden. Adults aged ≥85 years experienced the highest crude mortality rates and the steepest long-term increase, while mortality also rose markedly among those aged 75–84 years. Aging is accompanied by loss of skeletal muscle, reduced appetite, impaired mobility, and declining physiological reserve, all of which may be exacerbated by CKD-related metabolic and inflammatory disturbances. Frailty is highly prevalent among patients with CKD and kidney failure and has been associated with a substantially increased risk of mortality (23). Older adults also carry a greater burden of cardiovascular disease, diabetes, polypharmacy, infection susceptibility, and functional impairment. Consequently, malnutrition may interact with kidney dysfunction and multimorbidity to produce a state of heightened vulnerability in which relatively modest clinical stressors precipitate hospitalization, functional decline, or death. The exceptionally steep increase among adults aged ≥85 years suggests that population aging may become an increasingly important driver of CKD- and malnutrition-related mortality in the United States.

The forecasting analysis provides a forward-looking perspective on this burden. ARIMA models suggested continued increases in both AAMRs and annual deaths through 2048. These projections should be interpreted cautiously because time-series models extrapolate historical patterns and cannot fully incorporate future changes in demographic structure, therapeutic innovation, nutritional management, diagnostic practices, or health policy. The widening prediction intervals with longer forecast horizons further illustrate this uncertainty. Nevertheless, the consistently upward central trajectories suggest that the burden is unlikely to decline spontaneously if recent patterns persist. From a public health and health-system perspective, continued growth in absolute deaths could translate into increasing demand for nephrology services, geriatric care, nutritional support, primary care, and long-term care as the U.S. population ages.

The present findings have important clinical and public health implications. Nutritional risk should be recognized earlier in the course of CKD rather than being addressed only after overt weight loss or advanced PEW has developed. Current clinical guidelines recommend systematic nutritional screening, comprehensive nutritional assessment, and individualized medical nutrition therapy as integral components of CKD management (13). In practice, assessment should not rely exclusively on body weight or serum albumin but, when feasible, should incorporate recent weight change, dietary intake, muscle mass and strength, functional status, and clinical evidence of PEW or frailty. Particular attention may be warranted for populations identified in the present study as having high or rapidly increasing mortality, including older adults, males, NH Black adults, and residents of nonmetropolitan communities. At the same time, effective nutritional management requires more than individual counseling. Social determinants of health substantially influence CKD outcomes and patients’ ability to engage in recommended care (39), while safety-net healthcare systems frequently serve populations facing disproportionate disease burden and barriers to guideline-concordant management (40). Improving access to renal dietitians, nephrology and primary care services, multidisciplinary CKD management, telehealth, transportation, and affordable nutritious food may therefore facilitate earlier recognition of nutritional deterioration, strengthen continuity of care, and reduce disparities. From a public health perspective, linking nutritional surveillance with efforts to address food insecurity and unequal healthcare access may help translate the demographic and geographic disparities identified in this study into actionable prevention and resource-allocation priorities.

Importantly, the mortality rates reported in this study represent deaths in which both CKD and malnutrition were documented on the death certificate. They should not be interpreted as estimates of the prevalence of malnutrition among individuals with CKD or as evidence that malnutrition directly caused each death. Nutritional disorders may be under-recognized or omitted from death certificates when cardiovascular disease, infection, or kidney failure dominates the terminal clinical presentation. Consequently, the absolute mortality burden identified in this analysis may be conservative. Conversely, increasing awareness and documentation of malnutrition over time could have contributed to some of the observed increase. The principal value of this study therefore lies in characterizing long-term patterns and disparities within a consistent national mortality surveillance framework rather than establishing individual-level causal relationships.

### 4.1 Limitations

Several limitations should be acknowledged. First, this study relied on death-certificate data and was therefore subject to misclassification and incomplete ascertainment of both CKD and malnutrition. Malnutrition may be particularly under-recorded as a contributing cause of death, potentially leading to underestimation of the true mortality burden. Second, CDC WONDER death certificate data provide information on causes of death but lack individual-level clinical details regarding disease severity, CKD stage, dialysis status, nutritional status, treatment history, and the temporal sequence between CKD, malnutrition, and death. Each death certificate contains a single underlying cause of death and may include up to 20 additional multiple causes of death (25). Therefore, documentation of CKD or malnutrition as a contributing cause indicates that the condition was recorded on the death certificate but does not necessarily indicate that it was severe, the primary driver of death, or temporally antecedent to the other condition. Accordingly, the observed relationships should be interpreted as population-level associations, and causal relationships cannot be inferred from these data. Third, changes in disease recognition, coding practices, and death-certificate documentation over the 26-year study period may have influenced observed temporal trends. Fourth, race and ethnicity categories were harmonized across the bridged-race and single-race CDC WONDER classification systems. Although this approach improved longitudinal consistency, the two classification systems are not fully equivalent, particularly with respect to individuals reporting more than one race. Therefore, residual discontinuity in race-specific estimates across the classification transition cannot be excluded. In addition, combining several smaller racial populations into the NH other racial group may obscure potentially important subgroup-specific differences. Fifth, urban–rural data were available only through 2020, preventing evaluation of more recent changes in these disparities. Sixth, ARIMA models assume that historical temporal structures remain informative for future patterns and cannot account for unforeseen demographic, therapeutic, healthcare, or policy changes; therefore, long-term forecasts should be interpreted as indicative trajectories rather than precise predictions. Finally, variation in cause-of-death documentation across populations and healthcare systems may have influenced the magnitude of the observed demographic and geographic disparities.

## 5 Conclusion

In this nationally representative study of mortality trends from 1999 to 2024, mortality involving both CKD and malnutrition increased substantially among U.S. adults aged ≥45 years, with a sustained acceleration after 2014. The burden was disproportionately high among males, older adults, particularly those aged ≥85 years, NH Black adults, and nonmetropolitan populations during the available 1999–2020 urban–rural observation period, while NH White adults experienced the most rapid long-term increase. Forecasting analyses further suggested that both mortality rates and annual deaths may continue to rise if recent trends persist. These findings highlight the need to integrate early nutritional risk identification and longitudinal nutritional assessment into routine CKD care, while prioritizing high-risk and underserved populations. Strengthening multidisciplinary kidney and nutritional care and addressing disparities in healthcare access and food security may be important for reducing the future mortality burden associated with CKD and malnutrition.

## Data Availability

The original contributions presented in the study are included in the article/supplementary material, further inquiries can be directed to the corresponding authors.
